# Development and Application of a Novel pH-Responsive Bilayer Indicator Film for Yellowfin Seabream Preservation and Freshness Monitoring

**DOI:** 10.3390/foods14173019

**Published:** 2025-08-28

**Authors:** Shan Xue, Zhi Lin, Jia Liu

**Affiliations:** 1 College of Biological Science and Technology, Minnan Normal University, Zhangzhou 363000, China; lz32@mnnu.edu.cn; 2 Zhangzhou Food Science Research Institute, Zhangzhou 363000, China; 3 Research Institute of Zhangzhou-Taiwan Leisure Food and Tea Beverage, Zhangzhou 363000, China; 4 Guizhou Academy of Agricultural Sciences, Guiyang 550006, China

**Keywords:** pH-responsive, CS/FO-CAR/GEL/GSA/CUR, bilayer indicator film, yellowfin seabream preservation, freshness monitoring

## Abstract

A pH-responsive bilayer film was developed for real-time freshness monitoring and preservation of yellowfin seabream. The emulsified layer contained chitosan (CS) and flaxseed oil (FO), while the indicator layer comprised carrageenan (CAR), gelatin (GEL), grape seed anthocyanins (GSA), and curcumin (CUR). Optimization via response surface methodology determined the ideal formulation: CAR/GEL mass ratio 1.11:1, CS concentration 1.70%, and GSA/CUR dosage 53.99 mg/100 mL. The optimized film demonstrated superior mechanical properties (TS = 12.74 MPa, EAB = 68.24%), enhanced hydrophobicity (WVP = 1.21 × 10^−11^ g·m^−1^·s^−1^·Pa^−1^), and potent antioxidant activity (HRC = 92.35%). FTIR and SEM confirmed stable cross-linking and bilayer compatibility. Distinct color transitions (yellow → reddish-brown) occurred at different pH levels, correlating with fish spoilage indicators. During 25°C storage, the film effectively inhibited quality deterioration (TVB-N, TBARS, moisture loss, lipid oxidation) while extending shelf-life. Strong correlations were observed among TVB-N, TBARS, moisture (|r| > 0.97), and PUFAs’ spoilage contribution (r ≈ −0.99). This intelligent film enables dual-functionality: active preservation and visual freshness monitoring.

## 1. Introduction

The advanced capability of real-time food safety monitoring has positioned intelligent food packaging as a key research focus in recent years [1,2]. At present, both at home and abroad, there have been research reports on using plant pigments, such as purple sweet potato [3], blueberry anthocyanins [4], and red cabbage [5], as natural indicators for intelligent food packaging. Among them, grape seed anthocyanins (GSA), as an edible natural pigment with wide sources, excellent water solubility, and strong pH sensitivity [6,7], can be used as an indicator for intelligent food packaging. Anthocyanins rank among the most extensively studied bioactive natural compounds. As flavonoid derivatives, they demonstrate well-documented health-promoting properties including safety, antioxidant capacity, and protective effects against diabetes, obesity, cancer, microbial infections, and cardiovascular disorders [8]. Concurrently, their pH-dependent chromogenic behavior enables applications as colorimetric indicators for tracking metabolic pH shifts in foods or spoilage-induced pH changes mediated by microorganisms [8], which could enable real-time monitoring of packaged food safety while also delivering information on product response to environmental conditions and shelf-life determination [9,10].

This yellow diketone compound (CUR), naturally occurring in the rhizomes of *Zingiberaceae* and Araceae species, constitutes a rare botanical pigment [11]. Curcumin (Cur), a lipophilic diphenolic compound, serves as a natural food additive with coloring, flavoring, and spicing functions. Its recognized safety profile allows high-dose applications, while its bioactive properties—including antioxidant, anti-inflammatory, and antimicrobial effects—enhance its utility [12]. Notably, Cur’s chromatic transition from orange to red under alkaline conditions results from phenolate anion formation via hydroxyl group deprotonation, enabling its use as a pH-sensitive acid–base indicator [12,13]. Single natural pigments have relatively poor stability and functionality and certain limitations. Therefore, GSA can be mixed with CUR to make up for the instability of GSA and the limitation of the color change range of CUR. Zheng et al. [14] developed a mixed indicator label of anthocyanins and CUR in red cabbage for monitoring the freshness of pork. Zhou et al. [2] prepared a mixed bilayer indicator film of anthocyanin and CUR for monitoring the freshness of chicken in real time.

In studies such as the above-mentioned ones, since the exposure of meat packaging to storage can lead to a decrease in the stability of natural pigments such as anthocyanins and CUR, the pigments are usually dissolved in natural biopolymers to demonstrate their indicator functions [15]. In order to better fix natural pigments while considering the safety of food, biodegradable natural macromolecular polymers such as chitosan (CS), carrageenan (CAR), and sodium alginate are often selected as the film base materials for smart packaging films [16]. Among them, CS is a natural high-molecular polymer obtained by the deacetylation of chitin. It has good film-forming properties, biocompatibility, and biodegradability by itself and is mostly used in the preparation of bioedible membranes [17]. CAR has good film-forming ability. However, a single CAR film has poor mechanical strength and is hydrophilic. To improve the performance of the indicator film, CAR can be mixed with other biopolymers (such as CS, gelatin (GEL), etc.) for use to enhance its mechanical strength [18].

Plant-derived cold-pressed oils are recognized as functional enhancers that augment the bioactivity of biopolymeric packaging films [19]. Moreover, their inherent hydrophobicity impedes moisture transmission through packaging matrices [20]. Nanoscale encapsulation systems have garnered considerable research interest in food technology due to their distinctive advantages. These systems establish semi-permeable barriers that protect labile oil components against oxidation while enabling controlled release kinetics [21]. Concurrently, they promote oil droplet uniformity through nano-scale dispersion mechanisms. Flaxseed oil (FO) contains various active substances such as alpha-linolenic acid, phytosterols, vitamin E, and polyphenols. It has high nutritional value and functions such as antioxidation, anti-cancer, anti-inflammation, and blood pressure-lowering. It also has the functions of preventing and improving neurological and immune diseases, diabetes, and cardiovascular diseases, etc. [22] Tosif et al.’s [23] research showed that adding FO to the film can increase the moisture resistance of the film and improve its performance. Meanwhile, its emulsion can serve as an emulsified layer to enhance the performance of the film. Many scholars have confirmed [2,24,25] that the method of adding oil emulsification to the membrane can effectively improve the hydrophobicity and UV resistance of the membrane.

The yellowfin seabream (*Acanthopagrus latus*), a warm-water demersal fish species of the Sparidae family, is a commercially significant fish in the coastal regions of southeastern China. Although widely cultivated on a large industrial scale, its high moisture, protein, and fat content [26] make it highly susceptible to spoilage deterioration during storage, transportation, and marketing. This deterioration stems from endogenous chemical/enzymatic reactions and microbial contamination (including spoilage and pathogenic microorganisms), leading to significant declines in sensory and physicochemical quality. Consequently, developing preservation techniques and quality monitoring methods for yellowfin seabream has become imperative [26,27]. However, a review of the literature reveals that reports on the application of intelligent indicator films for preservation and freshness monitoring of yellowfin seabream remain scarce.

Based on this, the present study aimed to develop a novel pH-responsive bilayer indicator film with excellent mechanical strength and water resistance. The indicator layer was fabricated using CAR/GEL and GSA/CUR, while the outer layer was composed of a CS and FO blended emulsion. Through systematic investigation of the effects of CAR/GEL mass ratio, CS concentration, and GSA/CUR pigment mixture dosage on the film’s elongation at break (EAB), tensile strength (TS), water vapor permeability (WVP) and hydroxyl radical clearance (HRC), we optimized the preparation parameters to obtain the best-performing film. The optimally formulated film was subsequently characterized and applied for real-time freshness monitoring of yellowfin seabream (*Sparus latus*) stored at 25 °C. This study can offer practical guidelines for applying a pH-responsive bilayer indicator film to real-time fish freshness monitoring.

## 2. Materials and Methods

### 2.1. Material

The chitosan (CS, N-degree of deacetylation ≥ 90%, the relative molecular mass was about 50–90 kDa), gelatin, derived from fish scales (GEL, 99% purity, relative molecular weight 60 KDa to 130 KDa), carrageenan (CAR, 200 kDa to 1000 kDa), grape seed anthocyanins (GSA, polyphenol content > 95%), and curcumin (purity ≥ 95%) were all food grade and purchased from Zhejiang Yinuo Biotechnology Co., Ltd., Ningbo, China. The flaxseed oil (FO) (Arowana) (the content of linolenic acid is 48–70%) was purchased from RT-Mart Supermarket in Zhangzhou. The glycerin, chloroform, carbinol, anhydrous ethanol, salicylic acid, 2-thiobarbituric acid, ferrous sulfate heptahydrate, et al. were analytically pure and brought from Xilong Scientific Co., Ltd. (Shantou, China). Water was purified with a Milli-Q water purification system (Millipore Co., Ltd., Burlington, MA, USA). Fresh yellowfin seabream (*Sparus latus*) (about 0.6~1.0 kg weight) was purchased from RT-Mart (Zhangzhou, China) and transported to the laboratory alive.

### 2.2. Preparation of the CS/FO-CAR/GEL/GSA/CUR Bilayer Indicator Film

#### 2.2.1. Preparation of Mixed Pigment Solution

The mixed pigment solution was prepared according to the method of Zhou et al. [2]. A total of 0.8 g of GSA powder and 0.8 g of CUR powder were dissolved in 40 mL of 47.5% ethanol. The mixed pigment solution was stored in a dark room at 4 °C until use.

#### 2.2.2. Preparation of the Bilayer Indicator Film

Preparation of hydrophobic film: A certain mass concentration of CS was stirred in deionized water at 60 °C for 30 min by the magnetic stirrer (DF-101S, Shanghai Lichen Bangxi Instrument Technology Co., Ltd., Shanghai, China). Subsequently, FO (1% based on the deionized water content) was added to the CS solution. The mixture was homogenized for 5 min at 480 W and 20 kHz to form a blended emulsion, after which the pH of the emulsion was adjusted to 6.0 ± 0.1. Following degassing, 20 mL of the film-forming emulsion was cast onto a 10 cm diameter polytetrafluoroethylene (PTFE) plate and dried in a 50 °C air-drying oven for 6 h to obtain a hydrophobic single-layer film.

Preparation of indicator-layer film: A mixture of 5 g CAR and GEL powder was added to 300 mL of deionized water at 90 °C, along with 1% glycerol (based on the deionized water content), and stirred thoroughly at 90 °C and 1500 rpm for 0.5 h using a magnetic stirrer. After cooling the mixed solution to 60 °C, a predetermined mass concentration (mg/100 mL) of GSA/CUR mixed pigment solution was added and stirred until uniform, followed by pH adjustment to 6.0 ± 0.1. After defoaming, 30 mL of the solution was cast onto the aforementioned PTFE plate and further dried at 45 °C for approximately 6 h to form the bilayer film. Finally, the CS/FO-CAR/GEL/GSA/CUR bilayer indicator film in the PTFE plate was placed in a 25 °C drying oven for equilibration over 6 h before the film could be subjected to physicochemical property testing.

#### 2.2.3. Single-Factor Experiments

The effects of the CAR/GEL mass ratio (8:2, 7:3, 6:4, 5:5, 4:6, 3:7 g/g), CS concentration (0.5%, 1.0%, 1.5%, 2%, 2.5%, 3.0%), and GSA/CUR dosage (20, 30, 40, 50, 60, 70 mg/100 mL) on the elongation at break (EAB), tensile strength (TS), hydroxyl radical clearance (HRC), and water vapor permeability (WVP) of the CS/FO-CAR/GEL/GSA/CUR bilayer indicator film were systematically investigated.

#### 2.2.4. Response Surface Optimization Test

According to the results of single factor analysis, the effect of CAR/GEL mass ratio, CS concentration, and GSA/CUR dosage on the TS and EAB of the films was detected for further response surface test design in the predetermined range. The factor levels and the codes of response surface test design are shown in Table 1.

#### 2.2.5. Calculation of the Comprehensive Score of Film

The calculation of the comprehensive score of the CS/FO-CAR/GEL/GSA/CUR bilayer indicator film was based on the method of Jiang et al. [9]. Nine groups of experimental data were randomly selected from three groups of single-factor experiments. The data were standardized using SPSS 17.0 software, and the weights were obtained through factor analysis of dimension reduction processing. The positive effect indicators are referred to in Equation (1), the negative effect indicators are referred to in Equation (2), and the comprehensive score is referred to in Equation (3).

(1) $$P_{Positive\;effect}=\frac{R-Rmin}{Rmax-Rmin}$$

(2) $$P_{Negative\;effect}=\frac{Rmax-R}{Rmax-Rmin}$$

(3) $$S=a\times P_{1}+b\times P_{2}+c\times P_{3}+d\times P_{4}$$

where

P: Membership degree value;

Rmax: The maximum value in the determination of the index;

Rmin: The minimum value in the index determination;

S: Comprehensive score;

P_1_, P_2_, P_3_, P_4_: Membership degree values corresponding to the indicators;

a, b, c, d: The weights of the corresponding indicators.

### 2.3. Determination of Physical and Chemical Properties of Film

#### 2.3.1. The Thickness of the Film

The film was sectioned into specimens measuring 4 mm in width and 20 mm in length. Thickness measurements were taken at six randomly selected points across the film cross-section, covering upper, middle, and lower regions, using a digital micrometer (Mitutoyo BMD-25D) with an accuracy of 0.001 mm. The average value was reported as the final result.

#### 2.3.2. The Test of TS and EAB

The test of TS and EAB was referred to the method of XUE et al. [28]. Using a texture analyzer (CT3-10K, Brookfield AMETEK, Middleboro, MA, USA), tensile strength (TS) and elongation at break (EAB) were measured. Dumbbell-shaped film strips (30 mm × 10 mm) were clamped vertically between fixtures. A tensile test was initiated at constant speed until rupture, during which the initial gauge length (L0), rupture length (L1), and peak force (F) were recorded. Each sample was measured 3 times and averaged. The mechanical properties of the film were calculated according to Equations (4) and (5).

(4) $$S=\frac{F}{b\times d}$$

where

TS: tensile strength of the films, Mpa;

F: the maximum tension of the films, N;

b: width of the films, mm;

d: Thickness of the films, mm.

(5) $$EAB=\frac{L1-L0}{L0}\times 100$$

where

EAB: elongation at break of film, %;

L_1_—length of the film at fracture, mm;

L_0_—original length of film, mm.

#### 2.3.3. Determination of Hydroxyl Radical Scavenging Rate (HRC)

The hydroxyl radical scavenging rate (HRC) was measured according to the method of Xue et al. [28]. In a test tube, 0.2 mL of the membrane solution was added and dissolved with deionized water to a total volume of 12 mL, while 12 mL of distilled water was used as the blank group. To both the experimental and blank group tubes, 1 mL of FeSO_4_ solution (6 mmol/L) and 1 mL of H_2_O_2_ solution (6 mmol/L) were added and mixed well. Subsequently, 1 mL of salicylic acid solution (6 mmol/L) was added, followed by incubation in a water bath for 30 min to equilibrate. The absorbance was then measured at 510 nm. The HRC was calculated according to Equation (6).

(6) $$\mathrm{HRC}=\frac{A0-Ai}{A0}\times 100\%$$

where

Ai: Absorbance of the experimental group;

A0: Absorbance of the blank group.

#### 2.3.4. Water Vapor Permeability (WVP)

The measurement was performed according to the method of Mali [29]. First, 3 g of anhydrous calcium chloride was weighed into a small glass jar. An intact film was selected, its thickness measured using a vernier caliper, and then the film was securely fixed to cover the jar opening. After recording the initial weight, the sample was placed in a desiccator at 100% relative humidity. The weight was measured and recorded every 2 h. The WVP was calculated according to Equation (7)

(7) $$\mathrm{WVP}=\frac{\Delta m\times d}{t\times S\times \Delta P}\times 100\%$$

where

Δm: Weight difference before and after the 2-h interval (g);

d: Film thickness (cm);

S: Area of the bottle opening (cm^2^);

t: Duration of the interval (h);

ΔP: Water vapor pressure difference (kPa).

#### 2.3.5. FTIR Analysis

The CS/FO-CAR/GEL/GSA/CUR bilayer indicator film was trimmed to a suitable dimension (typically circular, 10–15 mm diameter) to guarantee flat, wrinkle-free surfaces devoid of contaminants. Against a blank reference background, samples were mounted on the holder and subjected to infrared transmittance measurements.

#### 2.3.6. SEM Analysis

Microstructural features of the CS/FO-CAR/GEL/GSA/CUR bilayer indicator were examined using scanning electron microscopy (Hitachi SU-8000, Tokyo, Japan) at 20 kV accelerating voltage. Post-drying, specimens were sectioned to optimal dimensions, adhered to stainless steel substrates with conductive carbon tape, mounted on copper stages for gold sputter-coating, and imaged to document surface/cross-sectional morphology [30].

### 2.4. Determination of pH Color Sensitivity of Film

The indicator film, packaged in transparent sealed bags, was placed in a desiccator maintained at a relative humidity of 50 ± 1%. Color measurements were taken every 1 day for a total of 10 days. To study the color sensitivity of the film, pH values of 1, 3, 5, 7, 8, 9, and 11 were selected. The indicator films were immersed in buffer solutions of the corresponding pH values, and their color changes were observed and recorded using a colorimeter after 6 min. The ΔE value was calculated according to Equation (8).

(8) $$∆E=\sqrt{{\left(L^{*}-{L_{0}}^{*}\right)}^{2}+(a^{*}-{a_{0}}^{*})({a^{*}-a)}^{2}+(b^{*}-{b_{0}}^{*}{)}^{2}}$$

where

L, a, b: Values of the film;

L_0_, a_0_, b_0_: Initial values, respectively.

### 2.5. The Application of Film in the Preservation and Freshness Detection of Yellowfin Seabream (Sparus latus)

The yellowfin seabream (*Sparus latus*) was humanely euthanized via immersion in an ice–water slurry (2.4 kg ice: 3.6 L water: 1.5 kg fish) for 20 min. Subsequently, specimens were promptly decapitated, descaled, eviscerated, and rinsed under tap water. All samples underwent ice storage ≤ 24 h prior to processing. Before experimentation, fish tissues were sectioned into 2 cm^3^ cubic portions. The fish meat (10 g per group) was put into triangular glass bottles and were sealed with film. The pH indicator layer of the film was downward. The fish preserved with the double-layer indicator film (comprising a hydrophobic layer and an indicator layer) served as the experimental group (EG), while the fish preserved with a single-layer film (without the hydrophobic layer) functioned as the control group (CG). The fish samples were stored at 25 °C for 0, 12, 24, 36, and 48 h, respectively. At each time point, changes in physicochemical indicators of the samples were measured, while corresponding color changes of the indicator film were observed and recorded.

#### 2.5.1. Determination of Moisture Content in Fish

A 5.0 g sample of fish was placed in a pre-dried weighing bottle (m_0_), and the initial weight (m_1_) was recorded. The sample was then placed in a drying oven at 105 °C for 2 h. After removal, the weighing bottle was cooled before the weight (m_2_) was weighed and recorded. The drying process was repeated (30 min per cycle after the first drying) until the weight difference between consecutive measurements was less than 2 mg. The moisture content was calculated according to Equation (9).

(9) $$\mathrm{Moisture}\;\mathrm{Content}=\frac{m_{1}-m_{2}}{m_{0}}\times 100\%$$

where

m_0_: Weight of undried sample (g);

m_1_: Initial mass of sample and the bottle (g);

m_2_: Constant mass of weighing bottle + sample after drying (g).

#### 2.5.2. Determination of pH of Fish

Weigh 10.0 g of fish meat sample and homogenize with 90 mL of distilled water using a blender. Allow the mixture to stand for 10 min, then filter the supernatant. Measure the pH value of the filtrate using a calibrated pH meter (Testo735-2, Testo AG, Titisee-Neustadt, Germany) at room temperature (25 ± 1 °C). Record triplicate measurements for each sample.

#### 2.5.3. Measurement of TVB-N

The TVB-N quantification was performed according to Xue et al. [31]. Samples (2.0 g) were treated with MgO (0.2 g) in a Kjeldahl distillation apparatus (Kjeltec 8400, FOSS, Hillerød, Denmark), followed by steam distillation analysis. Results were expressed as mg nitrogen per 100 g sample (mg N/100 g).

#### 2.5.4. Measurement of TBARS

The TBARS quantification followed Xue et al. [31]. Briefly, 5 g minced fish was homogenized with 50 mL of 7.5% trichloroacetic acid (TCA) containing 0.1% EDTA. The mixture was incubated at 50 °C (water bath) for 30 min, then filtered through Whatman No. 1 filter paper. A 3 mL aliquot of filtrate was reacted with 3 mL of 20 mmol/L 2-thiobarbituric acid (TBA) solution at 100 °C for 45 min. After cooling, the solution was centrifuged (5000× *g*, 4 °C, 10 min), mixed with 3 mL chloroform, and vortexed for 30 s. Following phase separation, supernatant absorbance was recorded at 532 nm and 600 nm against a blank (3 mL TCA + 3 mL TBA + 3 mL chloroform). Results were expressed as mg malondialdehyde (MDA) per kg sample according to Equation (10).

(10) $$\mathrm{TBARS}\;(\mathrm{mg}\;\mathrm{MDA}/\mathrm{kg})=\frac{c\times V}{m}\times 100\%$$

where

c: The concentration of malondialdehyde obtained from the standard curve/(μg/ mL);

V: Volume of sample liquid (mL);

m: Sample mass (g).

#### 2.5.5. Analysis of Fatty Acid Composition in Fish

The fatty acid composition of fish meat was analyzed through a standardized procedure involving lipid extraction followed by gas chromatographic determination [32]. For lipid extraction, 5.0 g of fish sample was homogenized with 60 mL of chloroform–methanol (2:1, *v*/*v*) and stirred at 45 °C for 2.5 h. After filtration, the extract was washed with 15 mL saturated NaCl solution in a separatory funnel, with the lower chloroform phase collected and dehydrated using anhydrous sodium sulfate. The purified lipid extract was obtained by rotary evaporation at 45 °C and quantitatively recorded. For fatty acid methyl ester (FAME) preparation, the extracted lipids were dissolved in benzene–petroleum ether (1:1, *v*/*v*) and derivatized with 14% BF_3_–methanol solution through 2.5 h incubation at 45 °C followed by 12 h refrigeration at 4 °C. The FAMEs were then extracted with n-hexane for subsequent GC analysis.

Chromatographic separation was performed on an SH-Rt™-2560 capillary column (100 m × 0.25 mm ID, 0.20 μm film thickness) using a temperature program starting at 140 °C (1 min hold), ramping at 4 °C/min to 240 °C (19 min hold). The GC system operated with nitrogen carrier gas at 20 mL/min, 10:1 split ratio, and 1 μL injection volume, with injector and detector temperatures maintained at 250 °C and 260 °C, respectively. Fatty acids were identified by comparing retention times with a 37-component FAME standard mixture and quantified using the area normalization method, with results expressed as relative percentages of total fatty acids.

### 2.6. Statistical Analysis

Statistical analyses were performed as follows: ANOVA with Tukey’s post hoc test (*p* < 0.05) using Microsoft Excel^®^ 2010 to determine inter-group differences; principal component analysis (PCA) executed in SPSS 17.0; optimization design implemented via Design-Expert 8.0.6; correlation analyses conducted with Excel^®^.

## 3. Results and Discussion

### 3.1. Results of Single Factor

#### 3.1.1. Effect of the CAR/GEL Mass Ratio on the Properties of the Bilayer Indicator Film

**Figure 1:**
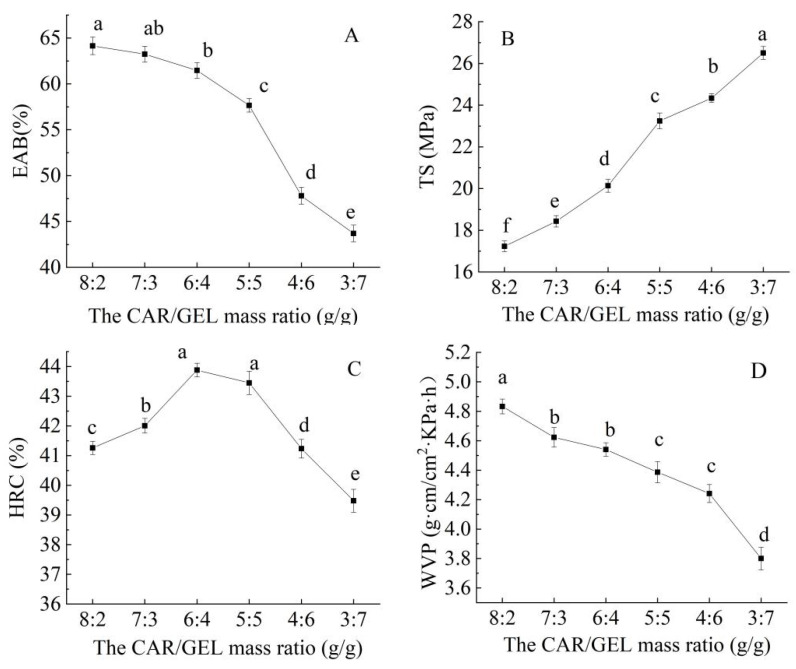
Effect of the CAR/GEL mass ratio on the elongation at break (EAB) (**A**), tensile strength (TS) (**B**), hydroxyl radical clearance (HRC) (**C**), and water vapor permeability (WVP) (**D**) of the CS/FO-CAR/GEL/GSA/CUR bilayer indicator film. (Different lowercase letters represent significant differences between data points (*p* < 0.05)).

As can be seen from the figures, the CAR (carrageenan)/GEL (gelatin) mass ratio significantly affected the properties of the bilayer film. As the CAR/GEL ratio in the film decreased (CAR reduced, GEL increased), the elongation at break (EAB) significantly decreased (*p* < 0.05) (Figure 1A), while the tensile strength (TS) significantly increased (Figure 1B). This may be because CAR molecules possess relatively rigid chains that can form a denser three-dimensional network structure, thereby enhancing the rigidity of the material. Films formed solely from CAR exhibited poor mechanical properties and brittleness, whereas the addition of GEL can effectively improve its performance [18].

The hydroxyl radical clearance (HRC) was higher when the CAR proportion was increased, possibly due to the presence of more active groups (e.g., sulfate esters) in CAR, which effectively scavenge free radicals [33]. In contrast, the HRC slightly decreased with an increase in the GEL proportion (Figure 1C).

The water vapor permeability (WVP) was higher when the CAR proportion was higher, which may be due to the hydrophilicity of CAR. When the GEL proportion increased, the WVP decreased (Figure 1D), as the dense structure formed by GEL effectively blocked water molecules [34].

Based on the trends observed in these indicators, when the CAR/GEL mass ratio was approximately 5:5 g/g, the film exhibited optimal performance, better meeting the requirements of food packaging for high strength and high barrier properties.

#### 3.1.2. Effect of the CS Concentration on the Properties of the Bilayer Indicator Film

**Figure 2:**
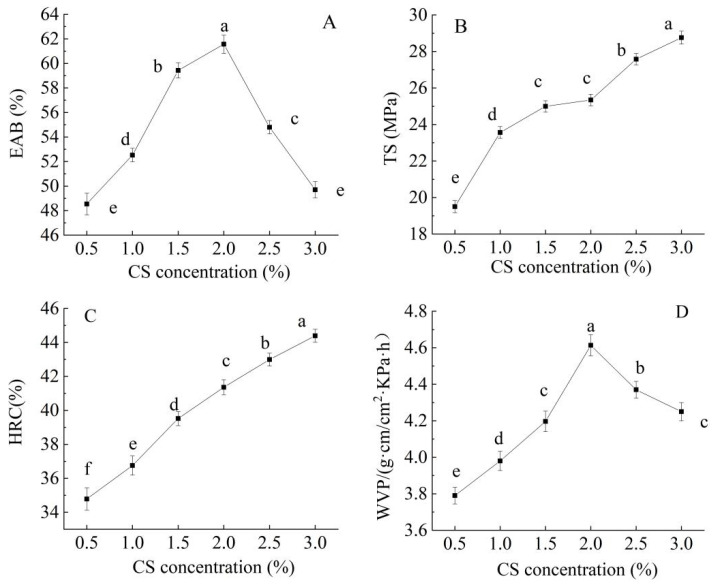
Effect of the CS concentration on the elongation at break (EAB) (**A**), tensile strength (TS) (**B**), hydroxyl radical clearance (HRC) (**C**), and water vapor permeability (WVP) (**D**) of the CS/FO-CAR/GEL/GSA/CUR bilayer indicator film. (Different lowercase letters represent significant differences between data points (*p* < 0.05)).

Figure 2A reveals that the EAB initially increased and then decreased with rising CS content, peaking at 1.5–2.0%. Low CS concentrations (≤1.5%) improved flexibility (as the flexible chains of CS compensated for the rigidity of CAR), whereas higher concentrations (>2.0%) led to increased brittleness due to excessive cross-linking [35].

As shown in Figure 2B, with the increase in CS content (0.5–3.0%), the TS significantly improved. This may be attributed to the abundant amino and hydroxyl groups in CS molecular chains, which can form additional hydrogen bonds and electrostatic interactions with CAR/GEL, thereby enhancing the cross-linking density of the film. Zhang et al. [36] reported that higher CS content leads to tighter intermolecular binding, resulting in increased TS but reduced toughness, which is consistent with the findings of this study.

The HRC significantly increased with higher CS concentrations (*p* < 0.05) (Figure 2C). This was because the free amino groups in CS effectively scavenged hydroxyl radicals, and higher CS concentrations provided more active groups, thereby enhancing antioxidant capacity [37].

The WVP initially increased and then decreased with increasing CS concentration (*p* < 0.05). This may be because CS possessed certain hydrophilicity, but the dense network structure formed with CAR at higher concentrations can hinder water molecule diffusion. Alternatively, it may form a more continuous barrier layer, thereby reducing the WVP [37] (Figure 2D).

Comprehensive analysis of these trends indicated that low CS concentrations (0.5–1.5%) effectively improved the flexibility of the film while moderately enhancing strength. In contrast, high CS concentrations (2.0–3.0%) significantly increase TS, HRC, and water resistance but compromise extensibility, making them suitable for high-strength packaging or antioxidant coatings. The optimal overall performance of the film is achieved when the CS content ranges between 1.5% and 2.0%.

#### 3.1.3. Effect of the GSA/CUR Dosage on the Properties of the Bilayer Indicator Film

**Figure 3:**
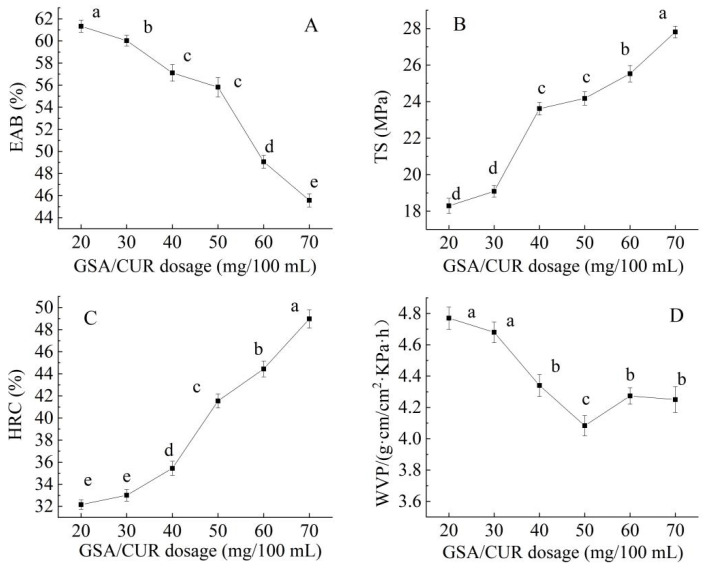
Effect of the GSA/CUR dosage on the elongation at break (EAB) (**A**), tensile strength (TS) (**B**), hydroxyl radical clearance (HRC) (**C**), and water vapor permeability (WVP) (**D**) of the CS/FO-CAR/GEL/GSA/CUR bilayer indicator film. (Different lowercase letters represent significant differences between data points (*p* < 0.05)).

As illustrated in Figure 3, the properties of the bilayer film exhibited significant variations with increasing GSA/CUR loading. Specifically, the EAB progressively decreased (*p* < 0.05) with higher additive concentrations (Figure 3A), likely attributable to the denser network structure induced by GSA/CUR incorporation, which consequently reduced film flexibility. Conversely, tensile strength (TS) demonstrated a positive correlation with additive content (Figure 3B), suggesting that GSA/CUR reinforcement enhanced the mechanical properties through potential formation of more stable crosslinked networks via interactions between GSA/CUR and the carrageenan/gelatin matrix [38].

Notably, the HRC showed marked improvement with increasing GSA/CUR loading (Figure 3C), indicative of the additives’ potent antioxidant capacity through effective free radical neutralization. The WVP initially decreased before exhibiting slight recovery at higher concentrations (Figure 3D). This biphasic trend may reflect initial obstruction of water diffusion pathways followed by potential structural heterogeneity at excessive loadings that compromises barrier performance. Such moisture-resistant characteristics are particularly valuable for food packaging applications, where enhanced water barrier properties contribute significantly to product stability and shelf-life extension [39].

Multivariate analysis of these functional parameters suggests that a GSA/CUR concentration of 50 mg/100 mL represents an optimal balance. At this loading level, the film maintains superior TS and HRC values, acceptable EAB retention, despite the expected reduction, and favorable WVP characteristics, indicating effective barrier properties. This formulation therefore achieves an optimal equilibrium between mechanical robustness, antioxidant activity, and functional flexibility, making it particularly suitable for advanced food packaging applications requiring multifunctional performance.

### 3.2. Analysis of the Comprehensive Score of Physicochemical Properties of the CS/FO-CAR/GEL/GSA/CUR Bilayer Indicator Film

#### 3.2.1. Suitability Test for Factor Analysis

The appropriateness of variables for factor analysis was evaluated using the Kaiser–Meyer–Olkin (KMO) metric and Bartlett’s test of sphericity. The KMO measure of sampling adequacy evaluated the adequacy of sampling by comparing the magnitudes of simple correlations to partial correlations among variables, primarily applied in multivariate statistics for factor analysis. Bartlett’s test examines whether the correlation matrix is an identity matrix, thereby testing the independence among variables. Therefore, these two tests must be conducted prior to factor analysis. The KMO statistic ranged between 0 and 1. A value closer to 1 indicated stronger inter-variable correlations and weaker partial correlations, suggesting better suitability for factor analysis. Generally, factor analysis was considered appropriate when the KMO statistic exceeded 0.5 [40].

Following the comprehensive scoring method for films described earlier, nine experimental datasets were randomly selected from three single-factor experiments to perform the suitability tests for factor analysis. As shown in Table 2, the KMO value was 0.524 (exceeding the threshold of 0.5 recommended by Kaiser) (Table 3), confirming that the comprehensive scoring model for physicochemical properties of films is suitable for factor analysis.

#### 3.2.2. Total Variance Explained

**Figure 4:**
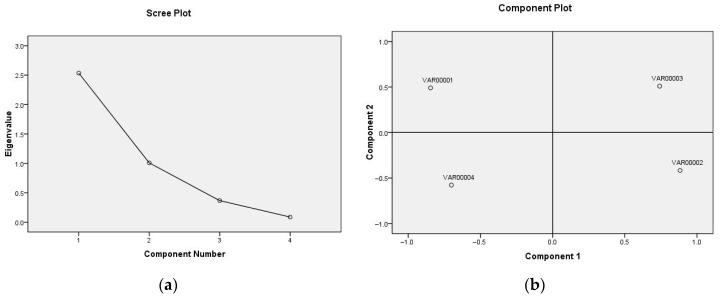
Gravel map of factor analysis (**a**) and the principal component distribution (**b**).

Using factor analysis in SPSS 17.0 software, factors with eigenvalues greater than 1 were extracted. The scree plot output (Figure 4a) shows that the eigenvalue curve contains two factors with eigenvalues exceeding 1. After varimax orthogonal rotation, the cumulative variance contribution rate of the four factors reached 88.59%, indicating that the two extracted factors accounted for 88.59% of the information from the original four indicators. The distribution of principal components is shown in Figure 4b, while the eigenvalues, contribution rates, and total explained variance of the relevant components are presented in Table 4. The eigenvectors of the first two principal components are shown in Table 5.

### 3.3. Response Surface Methodology (RSM) Optimization

#### 3.3.1. Establishment of Regression Model and Analysis of Variance (ANOVA)

The response surface experimental design plan and results was showed in Table 6. After regression analysis was performed, the quadratic polynomial regression equation describing the effects of CAR/GEL mass ratio (A), CS concentration (B), and GSA/CUR dosage (C) on the comprehensive score (Y) was obtained as follows:

(11) Y = 0.66 − 0.041A + 0.019B + 0.036C + 0.061AB + 0.075AC + 0.027BC − 0.054A^2^ − 0.091B^2^ − 0.054C^2^

Analysis of variance (ANOVA) for the response surface methodology (RSM) experiments (Table 7) revealed the following: The regression model was highly significant (*p* = 0.0002). The lack-of-fit term was non-significant (*p* = 0.0747), indicating good model fit. Factor A (CAR/GEL mass ratio), Factor C (GSA/CUR dosage), and interaction terms AB and AC showed highly significant effects on the comprehensive score (Y) (*p* < 0.01). Factor B (CS concentration) alone was not significant (*p* = 0.0575), but its interactions with A or C (AB, BC) approached significance (*p*-values close to 0.05). All quadratic terms (A^2^, B^2^, C^2^) were highly significant, demonstrating nonlinear relationships between the factors and comprehensive score (Y). Comprehensive analysis indicated the following order of factor influence on the comprehensive score within the tested range: A > C > B.

#### 3.3.2. Analysis of Interaction Effects and Optimization in RSM Experiments

**Figure 5:**
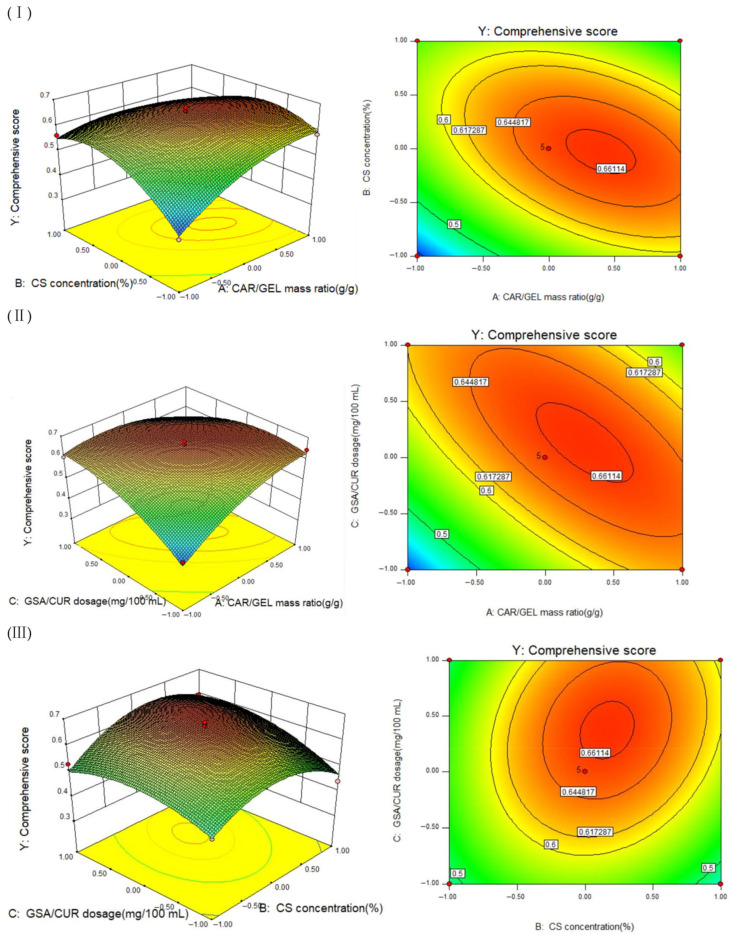
Three-dimensional response surface plots and contour plots depicting the pairwise interaction effects between CAR/GEL mass ratio (A) and CS concentration (B) (**I**); CAR/GEL mass ratio (A) and GSA/CUR dosage (C) (**II**); CS concentration (B) and GSA/CUR dosage (C) (**III**).

It can be known from Figure 5I that the slope of the isosurface of the interaction between A and B was steeper, indicating that the interaction between the mass ratio of CAR and GEL and the CS concentration was more obvious. When A was fixed, as B increased, the comprehensive score (Y) first increased and then decreased. When the mass ratio of CAR/GEL (A) was relatively high (close to 1) and the CS concentration (B) was moderate (0–0.5%), the Y value was relatively high, indicating that the rigidity of CAR needs to be synergistically optimized with the flexibility of CS. It can be known from Figure 5II that the interaction between A and C was obvious. When A was fixed, as C increased, the comprehensive score first rose and then decreased. When A high CAR/GEL ratio (A = 1) was combined with a moderate GSA/CUR dosage (C = 0.5–1 mg/100 mL), Y reached the peak, indicating that the compatibility of CAR with the active ingredient (GSA/CUR) was crucial to the performance. It can be known from Figure 5III that the slope of the surface where B interacted with C was relatively gentle, and the interaction effect between B and C was not significant. When the value of B was fixed, as the value of C increased, the comprehensive score first increased and then decreased. The interaction between B and C was relatively weak, which was consistent with the lower significance of BC in the analysis of variance.

#### 3.3.3. Determination and Verification of the Optimal Combination

After analysis and optimization by Design Expert V8.0.6.1, the optimal ratio of the indicator film was obtained as a mass ratio of CAR to GEL of 1.11: 1 (g/g), the CS concentration was 1.7%, and the GSA/CUR dosage was 53.99 mg/100 mL. Under this condition, the predicted comprehensive score was 0.6603. It was verified that under the optimal condition, the EAB of the membrane was 57.43 ± 3.51%, and the TS was 24.07 ± 1.25 MPa. The HRC was 46.61 ± 0.21%, and the WVP was 4.03 ± 0.08 10^−5^ g·cm/cm^2^·KPa·h. At this time, the comprehensive score was 0.6534 ± 0.0205, which was not significantly different from the predicted value (*p* > 0.05). Based on the above analysis, the prediction results of this model were relatively good.

### 3.4. SEM Analysis of the CS/FO-CAR/GEL/GSA/CUR Bilayer Indicator Film

**Figure 6:**
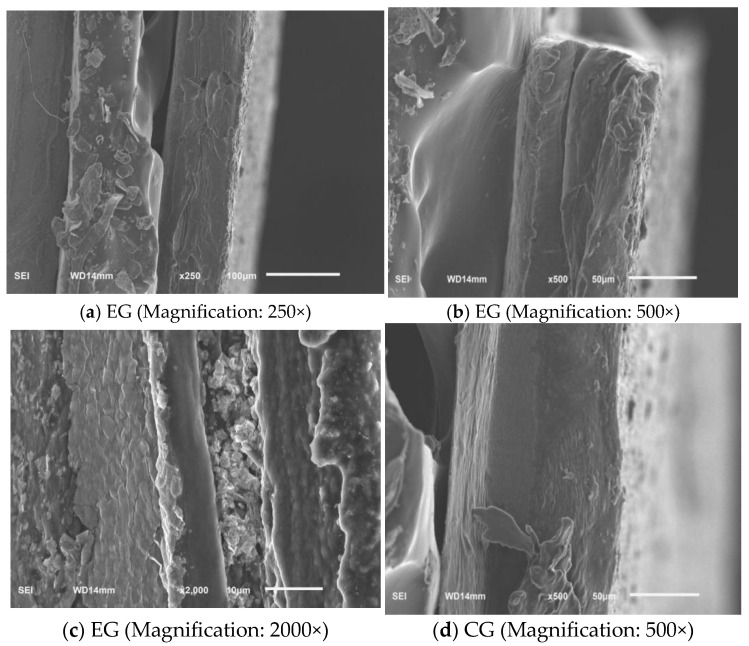
The SEM of the films (EG: the experimental group; CG: the control group).

The CS/FO-CAR/GEL/GSA/CUR bilayer indicator film was used as the experimental group (EG), and the monolayer film without CS/FO hydrophobic was used as the control group (CG). As can be seen from Figure 6a (Magnification: 250×), 6b (Magnification: 500×), 6c (Magnification: 2000×), the double-layer structure of EG can be clearly observed, and the combination was relatively tight with a relatively smooth surface, indicating that a compact double-layer indicator film had been successfully prepared. However, Figure 6d (Magnification: 500×) only had a single layer. In CG (single-layer film), uniformly distributed micropores can be seen, with obvious concavities and convexities on the surface, dominated by isolated spherical particles. In contrast, the porosity of the double-layer film was significantly reduced, presenting a smoother interface transition, and a dense network structure was formed between the particles.

### 3.5. Infrared Spectroscopy Analysis of the CS/FO-CAR/GEL/GSA/CUR Bilayer Indicator Film

**Figure 7:**
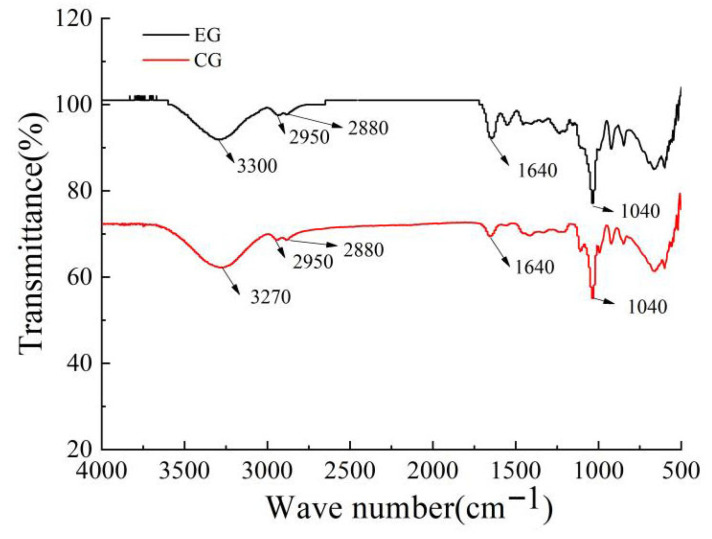
Infrared spectrum of films (EG: the experimental group; CG: the control group).

The absorption peaks at wavenumbers 3300 cm^−1^ and 2880–2950 cm^−1^ correspond to the stretching vibrations of O-H and C-H bonds, respectively. The peak at 1640 cm^−1^ represents the C=O stretching vibration of acetyl groups (Amide I), while the peak near 1040 cm^−1^ may be attributed to Amide II and C-C stretching vibrations [41]. As shown in Figure 7, the significantly enhanced C-H absorption peak of EG at 2880/2900 cm^−1^ and the significantly enhanced amide I/C=O absorption peak at 1640 cm^−1^ directly proved that the bilayer film (EG) contained components that were not presented in the monolayer film (CG)—namely, flaxseed oil rich in methylene and CS layers containing amide groups. Analysis of the intermolecular interaction changes in the samples revealed that the peak tip of CG was approximately 3270 cm^−1^, and that of EG was approximately 3300 cm^−1^. The O-H stretching vibration absorption peak of EG shifted towards higher wavenumbers, which may indicate that in the double-layer film structure, the strength of the hydrogen bond network formed by some hydroxyl or amino groups was lower than that of the single-layer film. Meanwhile, the peak of EG was wider than that of CG, and its intensity seemed to be slightly lower. This might be because the composite of the double-layer film introduced more components (CS, FO) and interfaces, which may lead to a more complex hydrogen bond environment with multiple hydrogen bond interactions of different intensities [42,43]. A slight decrease in intensity may have suggested a slight reduction in the proportion of O-H/N-H groups involved in the formation of strong hydrogen bonds (resulting in low wavenumber absorption) or partial substitution by other interactions (such as hydrophobic interactions). This was also consistent with the interpretation that the peak position was moving towards higher wavenumbers [44,45].

Additionally, the slight low shift of the polysaccharide characteristic peak near 1040 cm^−1^ strongly suggested that in the interface region of the bilayer film, ionic cross-linking (electrostatic interaction) had been formed between the sulfate groups of CAR and the amino groups of CS. This was the key molecular basis for the stability and functionality of the double-layer film structure. In addition, the peak position of the 1640 cm^−1^ amide I band showed no significant shift between EG and CG, indicating that the secondary structure of gelatin did not undergo large-scale overall changes after the formation of a bilayer film through compounding. The unchanged position of the C-H peak indicated that the alkyl chain environment was stable. The subtle changes in the polysaccharide fingerprint region suggested that the conformation of the sugar chain or local interactions may be affected by complexation.

### 3.6. Analysis of Color Stability and pH Sensitivity of Indicator Films

**Figure 8:**
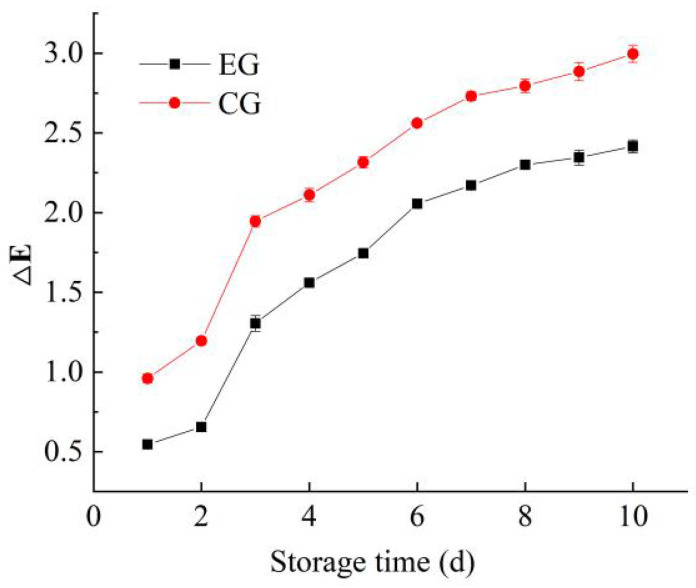
The color stability of films (EG: the experimental group; CG: the control group).

As shown in Figure 8, the ΔE value of the indicator films gradually increased with prolonged storage time. This phenomenon can be attributed to the degradation of the indicator GSA/CUR in response to environmental changes, leading to subtle color variations. Notably, the color changes occurred relatively rapidly in the initial stage but stabilized in later periods. Furthermore, the double-layer films with an emulsified layer (*CS/FO*) exhibited smaller ΔE value variations compared to single-layer films, indicating that the emulsified layer enhanced the stability of the indicator films. Within 10 days, all ΔE values remained below 5, which is imperceptible to the naked eye, demonstrating good color stability of the films. These findings align with the research of Chen et al. [42], who reported that composite anthocyanin and CUR systems exhibited higher sensitivity to meat freshness than individual anthocyanin or CUR indicators.

As evidenced by Table 8, the indicator film underwent a sequential color transition from bright yellow to reddish-brown and finally to dark red in buffer solutions with pH values ranging from 1 to 11. Under acidic conditions (pH < 8), the film exhibited negligible color changes, whereas pronounced chromatic shifts were observed under alkaline conditions (pH > 8). The GSA/CUR system combines the chromogenic properties of both GSA and CUR, but its behavior was predominantly governed by CUR [2]. The current research focuses on investigating two phytochemicals—curcumin and anthocyanins—as natural preservatives and colorants for active and intelligent macromolecular packaging materials. These phytochemicals exhibit pH-sensitive color changes, enabling them to function as freshness indicators in smart packaging systems [46]. For instance, under acidic conditions, anthocyanins display ruby red or pink hues, while curcumin maintains a distinct yellow color. Conversely, in alkaline environments, anthocyanins shift to blue or purple tones, whereas curcumin transitions to red or orange shades [47,48].

### 3.7. Application of Films in Yellowfin Seabream Preservation and Freshness Monitoring

#### 3.7.1. Changes in Moisture Contentof Fish

**Figure 9:**
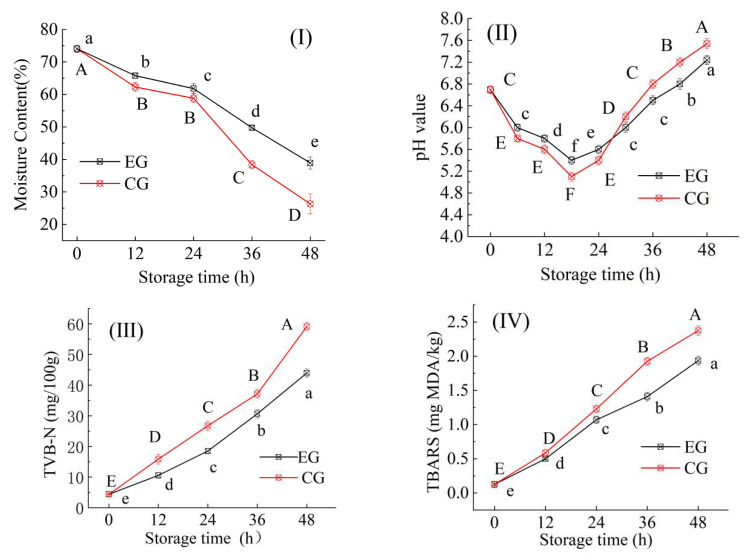
Changes in physical and chemical properties of fish preserved with different films (EG: the experimental group, presented in black lines; CG: the control group, presented in red lines. (**I**): water content; (**II**): pH; (**III**): TVB-N; (**IV**): TBARS). The a–f: different lowercase letters represented significant differences between EG data (*p* < 0.05); The A–F: different capital lettersdata represented significant differences between CG data (*p* < 0.05)).

Both EG and CG samples exhibited a progressive decline in moisture content during storage (*p* < 0.05), with EG demonstrating a significantly slower rate of moisture loss compared to CG (Figure 9I). This indicated that the emulsified bilayer structure more effectively impedes moisture migration from the fish substrate. The observed moisture retention effect was likely attributable to the incorporation of fish oil (FO) in the emulsified layer, which reduced water absorption and permeability. This finding aligns with prior studies on the barrier properties of tea oil-modified films [43], suggesting a generalized mechanism where lipid components enhance hydrophobicity and thus limit moisture transfer. Furthermore, the natural hydrophobicity of plant oils/cold-pressed oils may inhibit the adsorption and penetration of water by the packaging film, which was consistent with the results of this study.

#### 3.7.2. Changes in the pH Value of Fish

The effect of different films on the pH value of fish meat during storage at 25 °C is shown in Figure 9II. Compared with the control group (CG, monolayer film), the experimental group (EG, bilayer film) exhibited a slower pH decline rate, and the pH began to rise after 24 h of storage, whereas the pH of the CG reached its lowest point at 24 h before rebounding. This indicated that the bilayer film more effectively mitigated the acidification of fish meat in the early stage, likely due to its superior barrier properties, which reduced oxygen and microbial infiltration, thereby inhibiting oxidation and microbial activity in the fish [49]. Additionally, the bilayer film may help retain moisture in the fish meat, slowing the impact of water evaporation on pH [49]. Overall, the bilayer film demonstrated better performance in delaying acidification and maintaining fish meat quality, while the monolayer film accelerated acidification, negatively affecting freshness and taste. In a similar study, Takma and Korel [50] fabricated active polyester films enriched with antimicrobial coatings of CH and alginate containing black cumin oil and evaluated the effect of the active packaging film on the quality and shelf life of chicken breast meat at 4 °C. A greater increase in pH values in control samples were reported, and the active film containing black cumin oil was identified as susceptible to maintain the safety and quality of chicken meat.

#### 3.7.3. Changes in the TVB-N Value of Fish

The total volatile basic nitrogen (TVB-N) content serves as a key indicator for evaluating proteinaceous product spoilage, primarily consisting of volatile nitrogenous compounds derived from amine degradation [51,52]. As shown in Figure 9III, the TVB-N value of fish meat increased significantly (*p* < 0.05) with prolonged storage time, with the control group (CG) exhibiting a faster and greater increment compared to the experimental group (EG). According to GB 2733-2015, fish meat is considered inedible when the TVB-N value exceeds 20 mg/100 g [53]. At 25 °C, the CG approached this regulatory threshold after 24 h of storage, and both groups surpassed 20 mg/100 g by 36 h. Notably, the CG demonstrated a more rapid TVB-N increase than the EG during later storage stages, indicating that the emulsified indicator film effectively delayed TVB-N accumulation in fish meat. This result was similar to the research conclusions of Ji et al. [19], Saadat et al. [54], and Gursoy et al. [55]

#### 3.7.4. Changes in TBARS Value of Fish

The thiobarbituric acid reactive substances (TBARS) value is a widely used metric for lipid oxidation, where higher values correlate with greater oxidative deterioration and reduced product quality. Figure 9IV reveals that both EG and CG exhibited significant increases in TBARS values (*p* < 0.05) over time. After 48 h of storage at 25 °C, the CG reached a TBARS value of 2.39 mg MDA/kg, while the EG consistently showed lower values. These results demonstrated that the CS/FO-CAR/GEL/GSA/CUR bilayer composite indicator film effectively mitigated lipid oxidation in fish meat. The deterioration of meat quality was largely attributed to lipid and protein oxidation. During storage, lipid oxidation was commonly evaluated via the TBARS method, which quantified malondialdehyde (MDA) as a terminal oxidation product [56]. According to Ji et al., the CS film based on tea seed oil nano-microcapsules was more effective at reducing the total bacterial count, TBARS, and TVB-N values of pork [19], which is consistent with the results of this study.

#### 3.7.5. Changes in the Fatty Acid Composition of Fish

This study analyzed changes in the fatty acid composition of fish preserved with different films during 48 h storage at 25 °C (Table 9). The results demonstrated that the bilayer composite film (EG) more effectively maintained fatty acid stability compared to the monolayer film (CG). Experimental data revealed that the EG showed significantly slower accumulation of saturated fatty acids (SFA) (39.6% at 48 h vs. 43.08% in CG), while monounsaturated fatty acids (MUFA) in EG exhibited an initial increase peaking at 36.54% at 24 h, followed by a decline, contrasting with the continuous decrease observed in CG. Notably, the EG provided superior protection for polyunsaturated fatty acids (PUFA), with a higher retention rate (27.72% at 48 h) than CG (25.12%). The loss rates of key ω-3 fatty acids (EPA and DHA) in EG were 16.2% and 4.9% lower, respectively, compared to CG. This protective effect was attributed to the synergistic mechanisms of the bilayer film: its dense structure effectively blocked oxygen permeation, while active components (e.g., GSA/CUR) scavenged free radicals, inhibiting lipid oxidation chain reactions [57,58]. The findings indicated that the CS/FO-CAR/GEL/GSA/CUR bilayer composite film significantly delayed lipid oxidation through dual physical barrier and chemical antioxidant mechanisms, offering an effective preservation solution for fish rich in unsaturated fatty acids, with substantial practical implications. These results aligned with the TBARS value changes observed in Section 3.7.4.

#### 3.7.6. Monitoring of Fish Freshness

**Figure 10:**
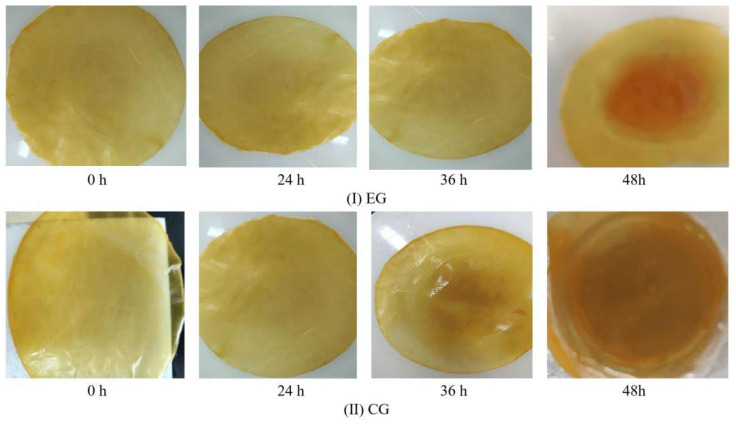
The color change of films ((**I**): EG; (**II**): CG).

The color changes of indicator films for monitoring fish freshness are shown in Figure 10. During the 48 h storage period, both EG and CG exhibited more pronounced color variations in later stages compared to initial phases. Notably, the EG demonstrated more uniform color transitions, with a distinct progression from yellow to reddish-brown after 36 h. At this time point, the ΔE value of EG measured 9.15 ± 0.95, reaching 15.14 ± 1.26 by 48 h. These chromaticity measurements correlated with the data in Table 8 and aligned with the aforementioned physicochemical parameter trends, confirming that the CS/FO-CAR/GEL/GSA/CUR bilayer indicator film effectively monitored fish freshness through visible color changes corresponding to spoilage status. While current applications of natural pH-responsive macromolecular polymers in fresh meat detection predominantly employ single-indicator monolayer films [59,60,61], the dual-indicator bilayer film developed in this study demonstrated superior performance characteristics. Comparative analysis revealed our innovative film not only achieved enhanced sensitivity but also maintained greater stability throughout the monitoring process. The multi-layered structure with compound indicators provided more reliable and distinguishable colorimetric responses, representing a significant advancement in intelligent packaging technology for real-time freshness assessment.

### 3.8. Correlation Analysis

**Figure 11:**
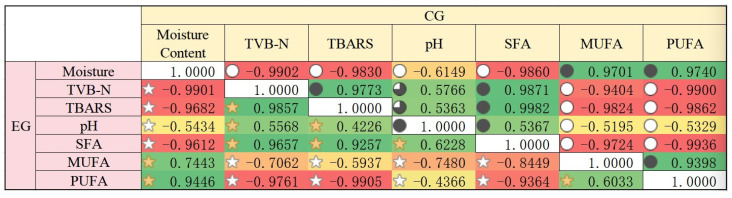
The correlation analysis of films (EG: the experimental group; CG: the control group).

Figure 11 presents the Pearson correlation coefficient matrices between different indicators during the storage of EG and CG, revealing the linear relationships among chemical indicators during food spoilage. Analysis of the EG showed that TVB-N exhibited extremely strong correlations with TBARS (0.986), PUFA (−0.976), and moisture content (−0.990), suggesting their synergistic changes during food spoilage. Moisture content was positively correlated with PUFA (0.945) but negatively correlated with SFA (−0.961), implying that moisture may influence fat oxidation or fatty acid composition. pH showed a significant negative correlation with MUFA (−0.748) but weaker associations with other indicators, possibly reflecting the specificity of microbial or enzymatic processes.

Analysis of the CG revealed that TVB-N and TBARS had an extremely strong positive correlation (r = 0.977), and both were highly negatively correlated with moisture content (r ≈ −0.99), indicating that protein decomposition and fat oxidation may occur synergistically alongside moisture loss. Notably, PUFA showed near-perfect negative correlations with TVB-N and TBARS (r ≈ −0.99), strongly suggesting that PUFA oxidation is a key driver of spoilage. SFA (saturated fatty acids) was positively correlated with spoilage indicators but significantly negatively correlated with MUFA (monounsaturated fatty acids, r = −0.972), reflecting dynamic changes in fat composition. pH exhibited weaker correlations with other indicators, showing only moderate associations.

These results provide a theoretical foundation for further studies, including validating the effects of different film preservatives on the physicochemical properties of fish during storage, constructing multivariate predictive models (e.g., PLS regression) to assess food freshness, and analyzing and comparing the correlations of key physicochemical indicators under different packaging conditions. Additionally, based on these findings, future research could delve deeper into the chemical and biological mechanisms by which films inhibit food spoilage, offering scientific support for targeted suppression of specific spoilage pathways.

## 4. Conclusions

This study optimized a pH-responsive bilayer film (CAR/GEL = 1.11:1, CS = 1.70%, GSA/CUR = 53.99 mg/100 mL) exhibiting superior mechanical strength, hydrophobicity, antioxidant activity, and color stability. The film effectively preserved yellowfin seabream by inhibiting quality deterioration (TVB-N, TBARS, lipid oxidation) while enabling real-time freshness monitoring via visible color transitions (yellow→reddish-brown) correlated with pH changes. Strong spoilage indicator correlations were identified (TVB-N-TBARS-moisture |r| > 0.97; PUFA degradation r ≈ −0.99). Beyond fish preservation, this intelligent film showed significant potential for monitoring perishable foods including meats, dairy, and aquatic products through pH-driven color signaling, offering dual-functionality in active packaging systems for extended shelf-life assurance.

## Figures and Tables

**Table 1:** The factor levels and the codes of response surface test design.

Factor	Levels		
	−1	0	1
A: The CAR/GEL mass ratio (m/m)	4:6	5:5	6:4
B: The CS concentration (%)	1.0	1.5	2.0
C: The GSA/CUR dosage (mg/100 mL)	40	50	60

**Table 2:** Test data of contribution rate by principal component analysis.

No.	EAB(%)	TS(MPa)	HRC(%)	WVP(10^−5^ g·cm/cm^2^·KPa·h)
1	47.80 ± 1.34	24.35 ± 0.14	41.05 ± 0.25	4.34 ± 0.08
2	63.53 ± 4.12	18.44 ± 0.13	42.33 ± 0.31	4.41 ± 0.49
3	57.65 ± 3.42	23.43 ± 0.12	43.24 ± 0.22	4.48 ± 0.08
4	60.05 ± 1.62	19.05 ± 0.25	33.16 ± 0.25	4.65 ± 0.09
5	49.15 ± 3.52	27.59 ± 0.09	36.94 ± 0.31	4.35 ± 0.49
6	45.62 ± 4.22	27.85 ± 0.16	49.05 ± 0.35	3.95 ± 0.33
7	55.32 ± 2.54	24.12 ± 0.26	41.69 ± 0.18	4.16 ± 0.24
8	54.98 ± 2.27	24.95 ± 0.16	44.26 ± 0.22	4.22 ± 0.08
9	61.29 ± 5.33	19.55 ± 0.08	39.97 ± 0.45	3.98 ± 0.07

**Table 3:** KMO and Bartlett’s test for dimensionality reduction models of texture index.

Kaiser–Meyer–Olkin Measure of Sampling Adequacy		0.598
Bartlett’s Test of Sphericity	Approx. Chi-Square	14.503
	df	6
	Sig.	0.024

**Table 4:** Eigenvalues and cumulative variance contribution rates of the related components.

Component	Eigenvalue	Variance Contribution Rate (%)	Cumulative Variance Contribution Rate (%)
Z_1_	2.535	63.373	63.373
Z_2_	1.009	25.217	88.59
Z_3_	0.368	9.198	97.789
Z_4_	0.088	2.211	100

Note: Only principal component factors with eigenvalues >1 were extracted.

**Table 5:** Eigenvectors of the first two principal components.

Component	EAB	TS	HRC	WVP
Z_1_	−0.845	0.883	0.742	0.701
Z_2_	0.491	−0.417	0.51	−0.578

**Table 6:** Response surface experimental design plan and results.

Test No.	A: CAR/GEL Mass Ratio (g/g)	B: CS Concentration (%)	C: GSA/CUR Dosage (mg/100 mL)	Y: Comprehensive Score
1	1	0	1	0.5338
2	0	−1	−1	0.4811
3	−1	1	0	0.5624
4	−1	0	1	0.6034
5	0	0	0	0.6445
6	0	0	0	0.6631
7	0	1	1	0.5964
8	0	1	−1	0.4392
9	1	−1	0	0.5826
10	−1	−1	0	0.3756
11	0	0	0	0.6781
12	0	0	0	0.6529
13	1	0	−1	0.6425
14	0	−1	1	0.5284
15	1	1	0	0.5238
16	0	0	0	0.6435
17	−1	0	−1	0.4132

**Table 7:** Analysis of response surface experiment variance.

Source of Variation	Sum of Squares	Degrees of Freedom	Mean Square	F-Value	*p*-Value	Significance
Model	0.13	9	0.015	25.65	0.0002	**
A	0.013	1	0.013	23.35	0.0019	**
B	0.002968	1	0.002968	5.15	0.0575	
C	0.010	1	0.010	17.74	0.0040	**
AB	0.015	1	0.015	26.16	0.0014	**
AC	0.022	1	0.022	38.75	0.0004	*
BC	0.003020	1	0.003020	5.24	0.0559	
A^2^	0.012	1	0.012	21.45	0.0024	**
B^2^	0.035	1	0.035	60.67	0.0001	**
C^2^	0.012	1	0.012	21.31	0.0024	**
Residual	0.004035	7	0.0005764			
Lack of Fit	0.003199	3	0.001066	5.10	0.0747	
Pure Error	0.0008360	4	0.0002090			
Total Deviation	0.14	16				

Note: ** indicates highly significant (*p* < 0.01); * indicates significant (*p* < 0.05).

**Table 8:** The color change of CS/FO-CAR/GEL/GSA/CUR bilayer indicator film under different pH.

pH	L*	a*	b*	∆E	Color
1	77.23 ± 0.25	10.52 ± 1.02	85.26 ± 0.55	3.77 ± 0.26	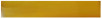
3	75.35 ± 0.32	11.22 ± 0.44	86.53 ± 0.29	5.12 ± 0.38	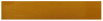
5	74.02 ± 0.11	11.56 ± 0.21	84.69 ± 0.25	4.56 ± 0.32	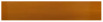
7	73.85 ± 0.22	12.46 ± 0.34	84.27 ± 0.67	4.34 ± 0.88	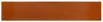
8	70.25 ± 0.56	16.34 ± 1.14	77.26 ± 1.54	15.03 ± 1.69	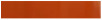
9	54.23 ± 2.24	41.63 ± 3.18	61.08 ± 5.63	39.58 ± 4.55	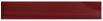
11	55.67 ± 1.94	25.34 ± 1.94	44.28 ± 7.44	19.92 ± 1.65	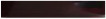

**Table 9:** Change of fatty acid composition of fish preserved with different films.

Fatty Acid Composition	EG					CG				
	0	12 h	24 h	36 h	48 h	0	12 h	24 h	36 h	48 h
C12:0	0.51 ± 0.06 ^e^	0.85 ± 0.02 ^c^	0.77 ± 0.01 ^d^	1.17 ± 0.01 ^b^	1.25 ± 0.02 ^a^	0.51 ± 0.06 ^dc^	0.6 ± 0.01 ^c^	1.11 ± 0.01 ^b^	1.17 ± 0.04 ^b^	1.26 ± 0.03 ^a^
C14:1n−9	0.2 ± 0.05 ^d^	0.44 ± 0.03 ^c^	0.94 ± 0.02 ^a^	0.73 ± 0.01 ^b^	0.47 ± 0.03 ^c^	0.2 ± 0.05 ^d^	0.28 ± 0.04 ^d^	0.78 ± 0.03 ^b^	0.57 ± 0.04 ^c^	1.38 ± 0.01 ^a^
C15:0	1.63 ± 0.17 ^a^	0.82 ± 0.02 ^c^	0.91 ± 0.02 ^b^	0.98 ± 0.03 ^b^	0.98 ± 0.02 ^b^	1.63 ± 0.17 ^a^	1.23 ± 0.02 ^b^	0.9 ± 0.04 ^c^	0.69 ± 0.01 ^d^	0.93 ± 0.01 ^c^
C15:1	0.33 ± 0.02 ^e^	3.26 ± 0.03 ^b^	3.04 ± 0.03 ^c^	2.53 ± 0.03 ^d^	3.67 ± 0.03 ^a^	0.33 ± 0.02 ^c^	0.28 ± 0.04 ^c^	2.09 ± 0.01 ^b^	3.56 ± 0.02 ^a^	3.53 ± 0.01 ^a^
C16:0	22.44 ± 0.29 ^d^	23.53 ± 0.04 ^c^	24.1 ± 0.03 ^b^	23.93 ± 0.01 ^b^	25.75 ± 0.03 ^a^	22.44 ± 0.29 ^d^	24.08 ± 0.03 ^c^	25.39 ± 0.03 ^b^	26.93 ± 0.02 ^a^	27.57 ± 0.03 ^a^
C16:1	5.4 ± 0.17 ^a^	3.77 ± 0.04 ^c^	2.06 ± 0.01 ^d^	4.3 ± 0.02 ^b^	3.82 ± 0.04 ^c^	5.4 ± 0.17 ^b^	6.86 ± 0.02 a	4.82 ± 0.01 ^c^	3.73 ± 0.01 ^d^	3.36 ± 0.02 ^e^
C18:0	5.36 ± 0.16 ^d^	6.1 ± 0.01 ^c^	6.66 ± 0.03 ^c^	7.15 ± 0.02 ^b^	9.47 ± 0.02 ^a^	5.36 ± 0.16 ^e^	6.2 ± 0.01 d	7.45 ± 0.01 ^c^	9.29 ± 0.04 ^b^	11.15 ± 0.02 ^a^
C18:1n−9	30.12 ± 0.27 ^a^	26.54 ± 0.04 ^b^	25.88 ± 0.04 ^d^	26.33 ± 0.11 ^c^	24.25 ± 0.01 ^e^	30.12 ± 0.27 ^a^	26.44 ± 0.02 b	25.5 ± 0.02 c	23.77 ± 0.02 ^d^	22.93 ± 0.04 ^e^
C18:3n−6	4.87 ± 0.17 ^a^	4.7 ± 0.04 ^b^	3.83 ± 0.02 ^c^	3.79 ± 0.04 ^c^	3.24 ± 0.04 ^d^	4.87 ± 0.17 ^a^	4.32 ± 0.02 b	4.91 ± 0.04 a	3.88 ± 0.03 ^c^	2.88 ± 0.01 ^d^
C18:3n−3	2.03 ± 0.0 ^c^	2.59 ± 0.03 ^b^	2.93 ± 0.02 ^a^	1.39 ± 0.01 ^d^	1.26 ± 0.01 ^e^	2.03 ± 0.07 ^c^	2.96 ± 0.03 c	2.16 ± 0.03 b	2.1 ± 0.04 ^b^	2.33 ± 0.04 ^a^
C20:1	0.2 ± 0.05 ^c^	0.27 ± 0.03 ^c^	2.44 ± 0.03 ^a^	0.43 ± 0.01 ^b^	0.14 ± 0.04 ^d^	0.2 ± 0.05 ^b^	0.19 ± 0.01 c	0.37 ± 0.04 a	0.12 ± 0.04 ^c^	0.31 ± 0.02 ^a^
C21:0	0.42 ± 0.02 ^ac^	0.38 ± 0.04 ^d^	0.25 ± 0.01 ^e^	0.62 ± 0.04 ^a^	0.5 ± 0.01 ^b^	0.42 ± 0.02 ^b^	0.54 ± 0.04 a	0.45 ± 0.03 b	0.36 ± 0.04 ^c^	0.47 ± 0.01 ^b^
C20:2	0.29 ± 0.01 ^d^	0.35 ± 0.02 ^c^	0.52 ± 0.04 ^a^	0.56 ± 0.03 ^a^	0.42 ± 0.01 ^b^	0.29 ± 0.01 ^b^	0.42 ± 0.03 a	0.31 ± 0.01 b	0.4 ± 0.01 ^a^	0.37 ± 0.04 ^a^
C22:0	0.82 ± 0.09 ^c^	1.42 ± 0.02 ^b^	0.33 ± 0.03 ^d^	1.43 ± 0.01 ^b^	1.65 ± 0.01 ^a^	0.82 ± 0.09 ^d^	0.94 ± 0.01 c	1.68 ± 0.01 a	1.55 ± 0.03 ^b^	1.7 ± 0.04 ^a^
C20:3n−6	3.53 ± 0.06 ^d^	5.93 ± 0.04 ^c^	3.86 ± 0.01 ^d^	6.04 ± 0.03 ^b^	6.32 ± 0.01 ^a^	3.53 ± 0.06 ^b^	4.63 ± 0.03 a	3.04 ± 0.01 c	2.72 ± 0.04 ^d^	2.14 ± 0.04 ^e^
C22:1	0.25 ± 0.05 ^c^	0.3 ± 0.02 ^b^	2.18 ± 0.03 ^a^	0.33 ± 0.01 ^b^	0.33 ± 0.04 ^b^	0.25 ± 0.05 ^da^	0.28 ± 0.04 a	0.25 ± 0.01 a	0.24 ± 0.02 ^a^	0.29 ± 0.02 ^a^
C20:3n−3	1.3 ± 0.07 ^d^	2.35 ± 0.02 ^b^	2.52 ± 0.03 ^a^	1.73 ± 0.02 ^c^	1.31 ± 0.02 ^d^	1.3 ± 0.07 ^d^	2.42 ± 0.03 c	1.39 ± 0.03 c	1.66 ± 0.03 ^b^	2.37 ± 0.04 ^a^
C20:4n−6	0.18 ± 0.02 ^d^	0.22 ± 0.04 ^c^	0.45 ± 0.04 ^a^	0.35 ± 0.04 ^b^	0.36 ± 0.02 ^b^	0.18 ± 0.02 ^b^	0.13 ± 0.02 b	0.4 ± 0.01 a	0.19 ± 0.04 ^b^	0.37 ± 0.04 ^a^
C22:2	1.16 ± 0.11 ^d^	2.03 ± 0.03 ^b^	2.74 ± 0.02 ^a^	1.91 ± 0.02 ^c^	1.04 ± 0.03 ^d^	1.16 ± 0.11 ^c^	2.12 ± 0.02 c	1.17 ± 0.01 c	2.27 ± 0.01 ^a^	1.43 ± 0.03 ^b^
C20:5n−3	10.84 ± 0.05 ^a^	6.62 ± 0.03 ^b^	6.38 ± 0.01 ^d^	6.69 ± 0.04 ^b^	6.54 ± 0.03 ^c^	10.84 ± 0.05 ^a^	7.16 ± 0.01 b	6.71 ± 0.02 c	6.65 ± 0.04 ^c^	5.63 ± 0.04 ^d^
C22:6n−3	8.13 ± 0.16 ^a^	7.53 ± 0.08 ^b^	7.21 ± 0.03 ^c^	7.61 ± 0.05 ^b^	7.23 ± 0.02 ^c^	8.13 ± 0.16 ^a^	7.92 ± 0.02 b	8.11 ± 0.03 a	8.15 ± 0.02 ^a^	7.6 ± 0.04 ^c^
SFA	31.18 ± 0.17 ^e^	33.1 ± 0.07 ^d^	33.02 ± 0.12 ^c^	35.28 ± 0.02 ^b^	39.6 ± 0.04 ^a^	31.18 ± 0.17 ^e^	33.59 ± 0.03 d	36.98 ± 0.04 ^c^	39.99 ± 0.03 ^b^	43.08 ± 0.01 ^a^
MUFA	35.5 ± 0.37 ^b^	34.58 ± 0.14 c	36.54 ± 0.14 ^a^	34.65 ± 0.23 c	32.68 ± 0.11 ^d^	35.5 ± 0.37 ^a^	34.33 ± 0.04 b	33.81 ± 0.03 ^c^	31.99 ± 0.04 ^d^	31.8 ± 0.02 d
PUFA	33.33 ± 0.29 ^a^	32.32 ± 0.24 ^b^	30.44 ± 0.12 ^c^	30.07 ± 0.14 d	27.72 ± 0.21 ^e^	33.33 ± 0.29 ^a^	32.08 ± 0.03 b	29.2 ± 0.03 ^c^	28.02 ± 0.04 ^d^	25.12 ± 0.02 ^e^

Note: The different lowercase letters in the EG and CG groups indicate significant differences between the data points (*p* < 0.05). EG: the experimental group; CG: the control group.

## Data Availability

The original contributions presented in this study are included in the article. Further inquiries can be directed to the corresponding author.
